# Supplementary material for: The interactome of intact mitochondria by cross-linking mass spectrometry provides evidence for coexisting respiratory supercomplexes
Source: Mol Cell Proteomics. 2017 Dec 8;17(2):216–32. doi: 10.1074/mcp.RA117.000470 (PMC5795388; doi:10.1074/mcp.RA117.000470)
Supplement: Supplemental Data [file supp_RA117.000470_133922_0_supp_23978_fzffwf.zip › spectra_annotation/mito_DR_spectra_annotation/421-1-2-1-8-1.pdf]

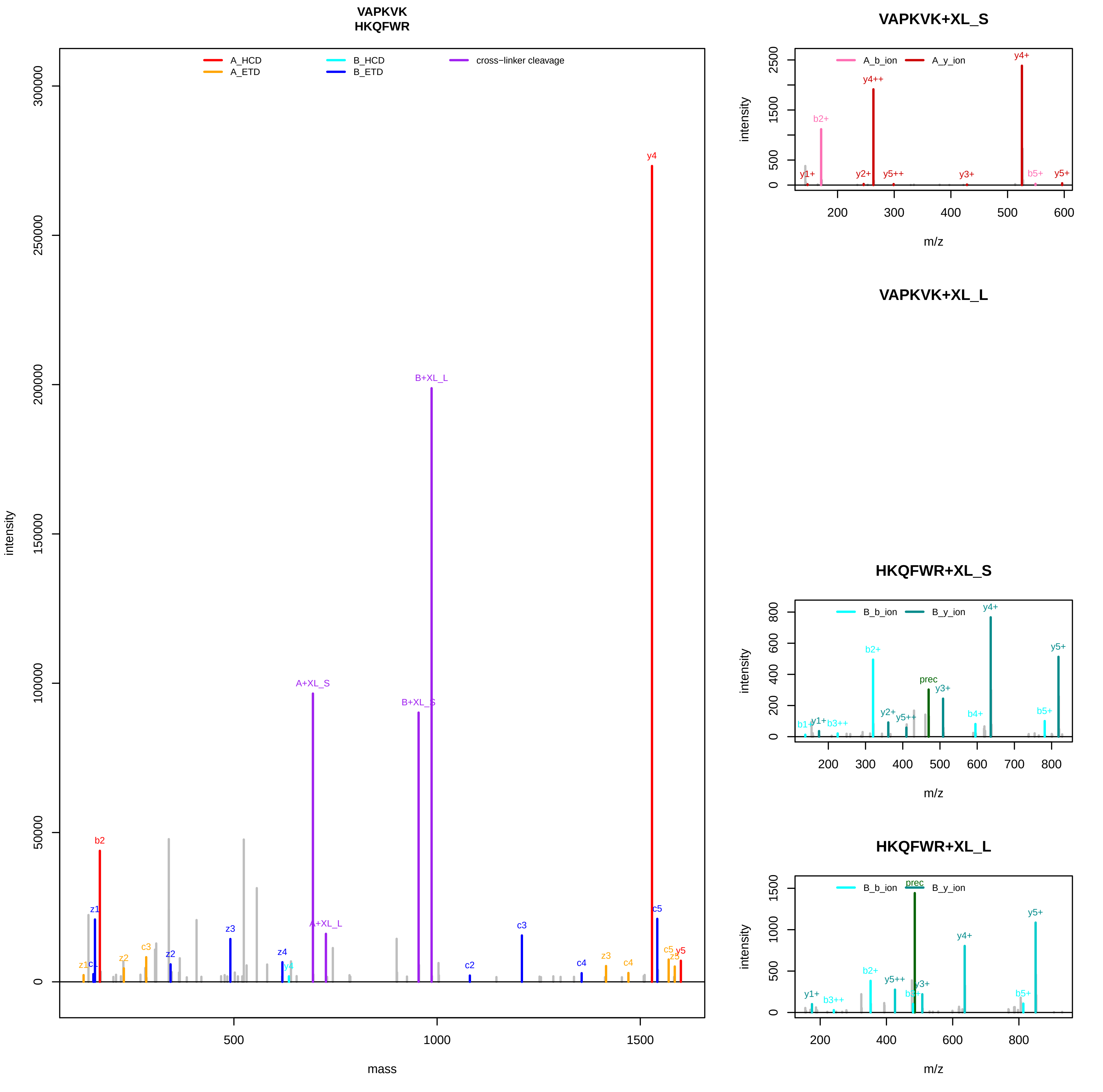

VAPKVK
HKQFWR
A_HCD
A_ETD
B_HCD
B_ETD
cross−linker cleavage
intensity
mass
0
50000
100000
150000
200000
250000
300000
500
1000
1500
b2
z1
c1
z2
c3
z3
z4
y4
A+XL_S
A+XL_L
B+XL_S
B+XL_L
c2
c4
c5
z5
y5
VAPKVK+XL_S
A_b_ion
A_y_ion
y1+
b2+
y2+
y4++
y5++
y3+
y4+
b5+
y5+
m/z
2500
1500
500
200
300
400
600
VAPKVK+XL_L
HKQFWR+XL_S
B_b_ion
B_y_ion
b1+
b3++
y5++
prec
b4+
700
800
HKQFWR+XL_L
b3+
